# Two levels vs. one level of phallopexy in the treatment of concealed penis in patients in pediatric age group

**DOI:** 10.3389/fped.2022.1001825

**Published:** 2023-01-20

**Authors:** Ahmed Elrouby, Israa Saad, Mostafa Kotb

**Affiliations:** Faculty of Medicine, Alexandria University, Alexandria, Egypt

**Keywords:** concealed, phallopexy, one level, two levels, buried

## Abstract

**Introduction:**

Concealed penis, which is the congenital type of buried penis, is a condition in which a normal-sized penis is totally or partially hidden by pubic, scrotal, or thigh skin. Several procedures had been described for its correction including phallopexy, that is, fixation of penile Buck's fascia to the sub-dermis.

**Aim of the work:**

Our work aims to study the difference in outcome between performing phallopexy at one level and at two levels.

**Material and methods:**

Our study included 180 uncircumcised patients who had a concealed penis while having an average length of an outstretched penis. These patients were divided into two groups: the first one was treated with one level of phallopexy at the 3 and 9 o'clock points, while the second group was treated with the same procedure in addition to another level of stitches at the mid-penile level. The follow-up was carried out for one post-operative year regarding penile skin edema, infection, congestion, necrosis, and/or re-retraction.

**Results:**

The overall success rate was 96.1% for a normally-looking penis without post-operative re-retraction. Re-retraction developed in two patients (2.2%) of those who had one-level phallopexy and in five patients (5.6%) of those who had two-level phallopexy without statistical significance (^FE^*p* = .444). Penile skin edema developed in 76 patients (42.2%) being significantly lower in patients with lower body weight (*p* = .030*).

**Conclusion:**

Phallopexy could be performed safely in the case of the concealed penis with satisfactory results. Two levels of phallopexy did not add any advantage to the post-operative results besides the fact that this may be demanding, time-consuming, and may require higher resources, so we recommend the easier one-level phallopexy in the treatment of such conditions with satisfactory results.

## Highlights

• What is currently known about this topic?

There is no consensus about the ideal procedure for dealing with pediatric patients with a concealed penis. Several techniques were described for the treatment of such conditions, including the excision of the dartos fascia, the lipectomy of suprapubic fat, and phallopexy or the fixation of the penile Buck's fascia to the subdermal level at the penile base, which could be performed at one or two levels. However, there were not many studies describing the difference in outcome between the two techniques, which was the aim of our study.

• What new information is contained in this article?

Phallopexy, or the fixation of the penile Buck's fascia to the subdermal layer in the case of the buried penis, has a high success rate of about 96.1%. There is no difference in the outcome when this procedure is performed at one level at the penile base or at two levels with the addition of an accessory stitch at the mid-penile level. Thus, the easier procedure of one level of phallopexy was recommended with satisfactory results in pediatric patients with a buried penis.

## Introduction

**Figure 1:**
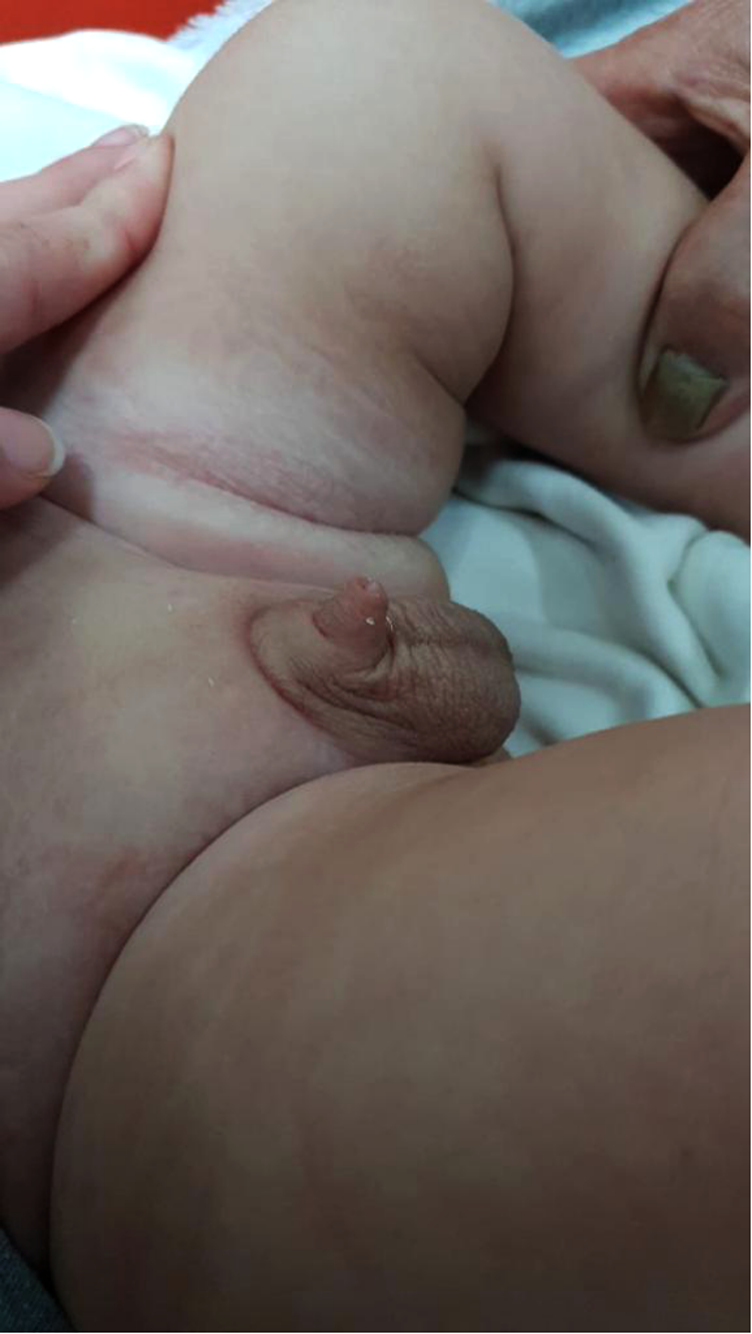
Concealed penis.

Concealed penis, which is the congenital type of buried penis, was defined for the first time by Keyes in 1919 as the following: “Absence of the penis exists when the penis, lacking its proper sheath of skin, lies buried beneath the integument of the abdomen, thigh, or scrotum” (1). The anomaly was classified by Crawford in 1977 (2) as partial or complete. Later, another classification was described by Maizels et al. in 1986 (3) as a buried penis caused by excess suprapubic fat or a lack of penile skin. Webbed penis, which occurs due to the presence of penoscrotal web, and trapped penis, in which the penile shaft is entrapped in the scarred fibrotic ring following trauma or circumcision (4) (Figure 1).

A possible explanation for a concealed penis is a firm tethering dartos tissue that pulls the penis inwards as well as the absence of normal attachment of the penile shaft to the skin dermis; this could be aggravated by the presence of excessive suprapubic fat (5). As a result of the inability to retract the prepuce backward with adequate cleaning, recurrent inflammation with scarring eventually develops, resulting in recurrent lower urinary tract infection and phimosis. Additionally, low self-esteem, depression, and painful erectile dysfunction can develop in adulthood (6).

Traditional circumcision for concealed penis patients can worsen the condition because removing the normal shaft skin instead of the foreskin, which does not attach to the underlying penile shaft, causes more concealment. Besides, the healing and narrowing of the incision line can bury the penis deeply. Correction of this situation by only redoing the circumcision can complicate the condition by requiring the removal of the remaining normal skin and requiring further grafting (7).

Therefore, surgical correction of the concealed penis should include dissection of the abnormally tethered dartos tissue in association with fixation of the penile Buck's fascia to the penile skin dermis. Despite that several procedures have been described in the literature by many authors for the correction of the concealed penis; no single operative technique could be performed for all cases (8).

The principle of penile fixation starts with complete degloving of the penis to the penile base and fixation of Buck's fascia to the penile dermis at the penile base to provide penile support and tether the penile skin, avoiding its re-retraction after circumcision (9).

## Aim of the work

Our work aims to study whether phallopexy (fixation of the penile skin dermis to Buck's fascia) performed in the case of the concealed penis should be performed at one level (at the penile base) or two levels (at the penile base and mid-penile shaft).

## Material and methods

Our retrospective study included 180 uncircumcised pediatric patients who presented to our institute with concealed penises from January 2019 to June 2020. Parents of the included patients complained mainly of the apparently small penile size, which is difficult to clean and causes psychological dissatisfaction. Patients with hypospadias, penile torsion, webbed penis, micro penis, congenital megaprepuce, and/or penile chordee were excluded from our study. All of the studied patients have a stretched penile length within the normal range for their ages, as described in the literature (10).

After a complete explanation of the privacy of patients' data and the possible publication of this manuscript, informed consent was signed by the patient's parents or caregivers.

Under general anesthesia; all of the studied patients were prepared by using povidone-iodine followed by backward retraction of the prepuce with complete cleaning and removal of smegma.

**Figure 2:**
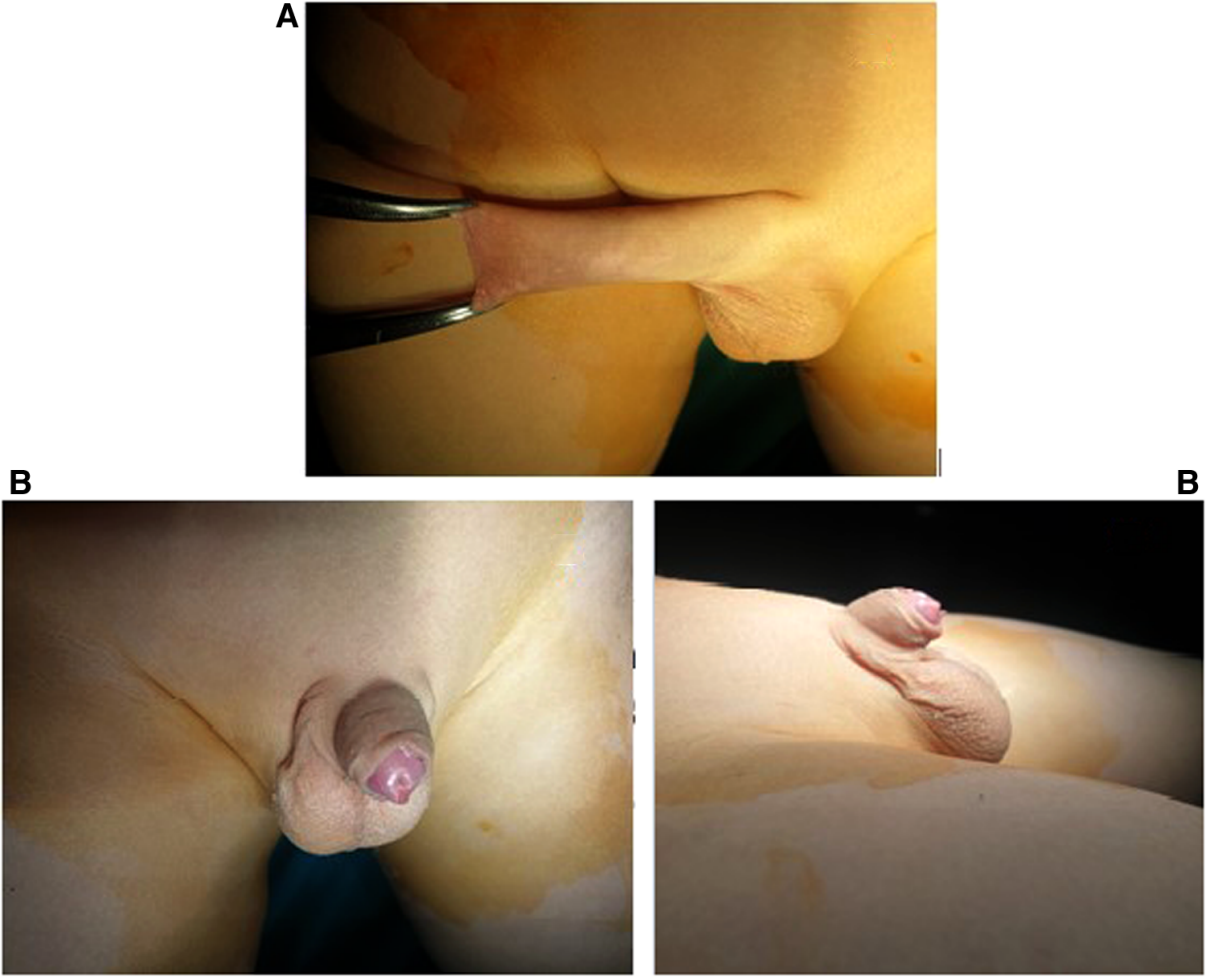
Circumcision with leaving around 5mm of mucosal collar.

Circumcision was started after the application of two retracting mosquito forceps at the mucocutaneous junction at 6 and 12 o'clock points leaving about 5 mm of the mucosal collar (Figures 2A,B).

**Figure 3:**
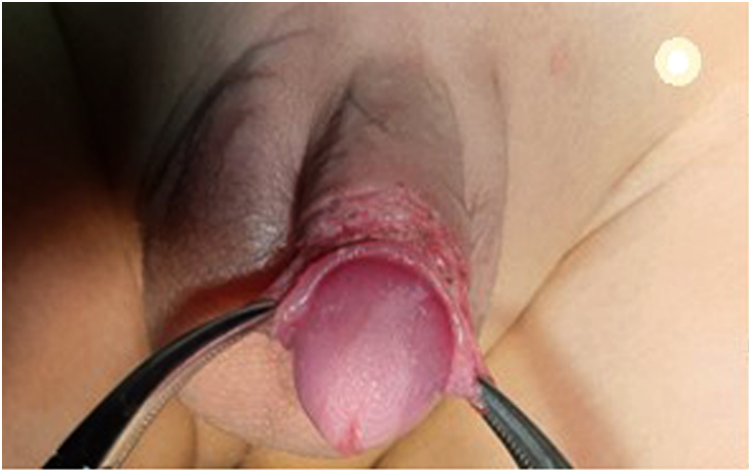
Two retracting mosquitos were applied at the sub-coronal level at 3 and 9 o'clock to pull the penis outward.

Two retracting mosquitos were applied at the sub-coronal level at 3 and 9 o'clock to pull the penis outward. Complete penile degloving with dissection and resection of the tethering dartos fascia down to the penopubic angle dorsally and the penoscrotal junction ventrally was performed (Figure 3).

**Figure 4:**
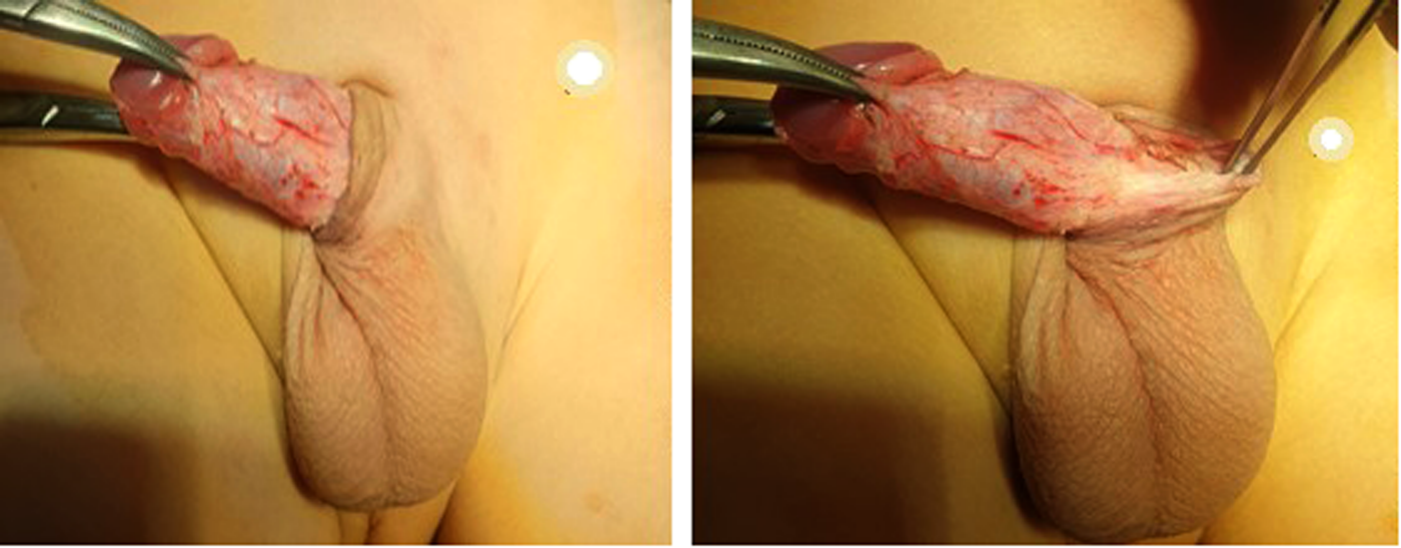
Degloving of the penis.

These forceps were kept in close alignment throughout the procedure, avoiding any degree of penile rotation (Figure 4).

**Figure 5:**
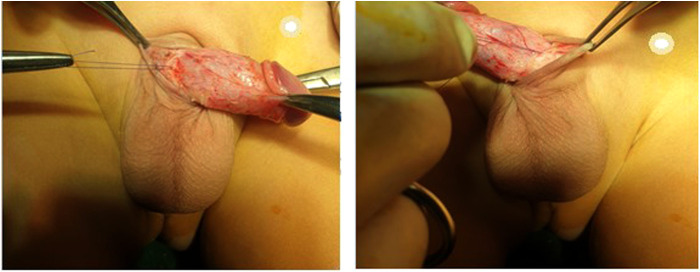
Penile fixation (phallopexy).

The procedure of penile fixation (phallopexy) was started by the application of two sutures at the penile base at 3 and 9 o'clock fixing, the penile dermis to Buck's fascia using PDS 5/0. This was the only step for patients with one level of phallopexy (Figure 5).

Similar stitches were applied distally at the mid-penile level at the same 3 and 9 o'clock points in patients with two-level phallopexy. These stitches were revised repeatedly during the procedure to avoid any post-operative skin dimpling. We selected 3 and 9 o'clock instead of 5 and 7 o'clock to allow more protection of the urethra, especially because two levels of phallopexy were performed in the second group.

**Figure 6:**
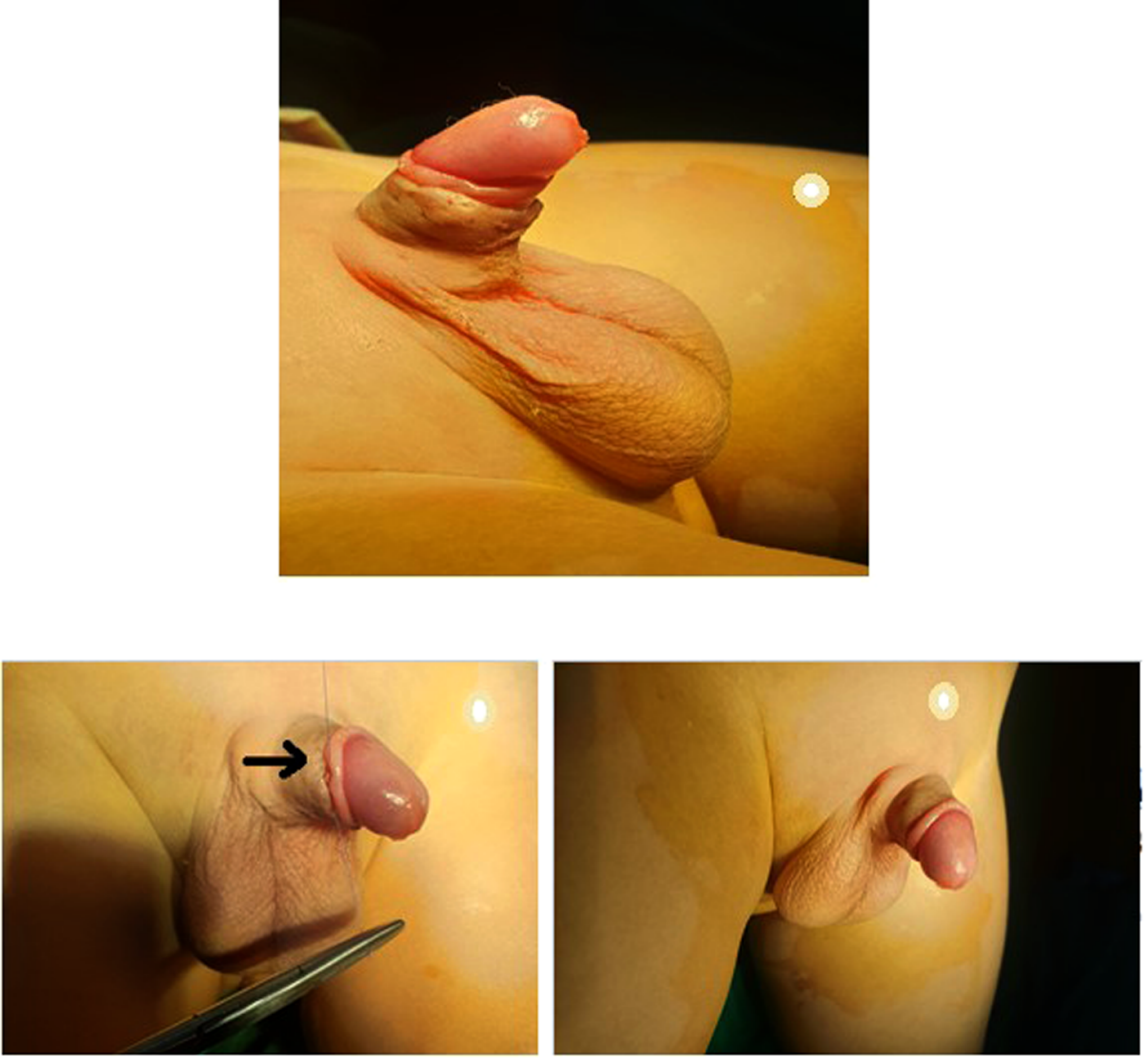
Suturing of the penile skin to the remaining mucosal collar using Vicryl 6/0 in a subcuticular fashion.

Removing the retracting forceps was followed by repositioning the penile skin over the penile shaft and suturing it to the remaining mucosal collar using Vicryl 6/0 in a subcuticular fashion (Figure 6). At the end of the procedure, a compression dressing with local antibiotic cream was applied over the wound. This dressing was then removed after 24 h followed by the repeated application of local antibiotic cream three times daily for 1 week, in addition to oral analgesic anti-inflammatory (NSAIDs), and anti-edematous for 5 post-operative days.

All of the studied patients were followed up at the end of the 1st post-operative week and then monthly for 1 year to detect the appearance of the penile skin with regard (congestion, necrosis), wound infection, edema, and/or re-retraction.

### Statistical analysis of the data

Data were fed to the computer and analyzed using the IBM SPSS software package, version 20.0 (Armonk, NY: IBM Corp). Qualitative data were described using numbers and percentages. The Kolmogorov–Smirnov test was used to verify the normality of the distribution. Quantitative data were described using range (minimum and maximum), mean, standard deviation, median, and interquartile range (IQR). The significance of the obtained results was judged to be at the 5% level.

The used tests were as follows

(1) Chi-square test

For categorical variables, to compare different groups.

(2) Fisher's exact

Correction for chi-square when more than 20% of the cells have an expected count of less than 5.

(3) Mann–Whitney test

For abnormally distributed quantitative variables, to compare two studied groups.

## Results

The age of the studied patients ranged between 3 months and 12 years, with a median of 13 months. Patients who had been subjected to two-level phallopexy were significantly younger than those who had one-level phallopexy (Table 1, *U* = 3232.0*, *p* = .019*).

**Table 1:** Comparison between the two studied groups according to different parameters.

	Total (*n* = 180)	One-level phallopexy (*n* = 90)	Two-level phallopexy (*n* = 90)	Test of significance	*p*
**Age (months)**					
Min. – Max.	2.11–144.0	4.0–129.0	2.11–144.0	*U* = 3232.0*	.019*
Mean ± SD	21.07 ± 26.79	26.01 ± 33.27	16.13 ± 16.94		
Median (IQR)	13.0 (8.0–22.0)	16.0 (9.0–24.0)	10.0 (7.0–18.0)		
**Weight (kg)**					
Min. – Max.	7.0–48.0	7.0–40.0	7.50–48.0	*U* = 2988.0*	.002*
Mean ± SD	12.27 ± 6.30	13.42 ± 6.99	11.19 ± 5.28		
Median (IQR)	11.1 (8.50–13.6)	12.0 (9.0–14.0)	9.0 (8.0–13.0)		
**Operative duration**					
Min. – Max.	20.0–45.0	25.0–45.0	20.0–45.0	*U* = 3491.5	.102
Mean ± SD	34.56 ± 7.16	35.50 ± 6.79	33.61 ± 7.42		
Median (IQR)	35.0 (30.0–40)	35.0 (30.0–40.0)	35.0 (30.0–40.0)		
Skin appearance	6 (3.3%)	0 (0%)	6 (6.7%)	*χ*^2 ^= 6.207*	^FE^*p* = .029*
Skin infection	1 (0.6%)	0 (0%)	1 (1.1%)	*χ*^2 ^= 1.006	^FE^*p* = 1.000
Edema	76 (42.2%)	32 (35.6%)	44 (48.9%)	*χ*^2 ^= 3.279	.070
Penile re-retraction	7 (3.9%)	2 (2.2%)	5 (5.6%)	*χ*^2 ^= 1.338	^FE^*p* = .444

*U*, Mann–Whitney test; *χ*^2^, Chi-square test; FE, Fisher exact.

*p*: *p*-value for comparing the studied groups.

*Statistically significant at *p* ≤ .05.

The body weight of the studied sample varied between 7 and 48 kg with a median of 11.1 kg. It was significantly lower in patients who had been subjected to two-level phallopexy (median = 9.0) kg than in those who had one-level phallopexy (median = 12.0) kg (Table 1, *U* = 2988.0*, *p* = .002*).

Recording the intra-operative surgical duration revealed that the median was 35 min ranging between 20 and 45 min with a mean of 34.56 ± 7.16 min. The difference in the surgical duration was not significant between one level and two levels of phallopexy (Table 1, *U* = 3491.5, *p* = .102).

Post-operative skin congestion in the form of dusky skin discoloration developed in six patients; all of them were in the group that had been subjected to two-level phallopexy; this was statistically significant. Penile dusky skin discoloration regressed gradually over the 1st post-operative month (Table 1, *χ*^2^ = 6.207*, ^FE^*p* = .029*).

Only one patient with two-level phallopexy developed a post-operative localized skin infection in the form of a pyogenic membrane with minimal oozing. Local antibiotic cream was continued for another week with oral antibiotics, resulting in complete resolution.

Post-operative penile skin edema had been observed in 76 patients (42.2%). A higher incidence was noted among those patients with two-level phallopexy (44; 48.9%) than in those with one-level phallopexy (32; 35.6%); however, this difference was not statistically significant (Table 1, *χ*^2^ = 3.279, ^FE^*p* = .070).

An observation noted among the studied patients was that post-operative penile skin edema developed more in those with larger body weights (Table 2, *U* = 3205.50*, *p *= .030*).

**Table 2:** Relation between edema with different parameters for the total sample (*n* = 180).

	Edema		*U*	*p*
	Absent (*n* = 104, 57.8%)	Present (*n* = 76, 42.2%)		
Age (months)				
Mean ± SD	15.74 ± 11.23	28.37 ± 38.03	3599.0	.305
Median (Min. – Max.)	13.0 (2.11–48)	13.50 (5–144)		
Weight (kg)				
Mean ± SD	10.96 ± 3.37	14.15 ± 8.51	3205.50*	.030*
Median (Min. – Max.)	9 (7–20)	12 (8–48)		
Operative duration				
Mean ± SD	33.99 ± 7.42	35.33 ± 6.75	3561.50	.247
Median (Min. – Max.)	35.0 (20–45)	35.0 (20–45)		

*U*, Mann–Whitney test.

*p*: *p*-value for comparing the studied groups.

*Statistically significant at *p* ≤ .05.

The main follow-up point of this operation, which is penile re-retraction, developed in only seven patients (3.9%) postoperatively. This was observed in two patients of those who had one level of phallopexy (2.2%) and in five patients of those who had two levels of phallopexy (5.6%); this difference was not statistically significant (Table 1, *χ*^2^ = 1.338, ^FE^*p* = .444).

## Discussion

Buried penis is defined as a normal-sized penis enclosed by one of the following layers: skin, subcutaneous tissue, and/or fat in the prepubic area. The lack of visibility of the penis makes the parents completely unsatisfied and worried about the future of his sexual life. Also the difficulty in penile cleaning of such patients' predisposes them to recurrent urinary tract infection. Another drawback of such a condition in some patients is avoiding difficulty and bad cosmoses which add to the parents' worry. The resulting phimosis may lead to recurrent urinary symptoms such as dribbling, spraying, and/or spraying, as well as repeated skin breakdown and maceration. Consequently, a delicate and timed management of such conditions should be performed to avoid the development of such complications (11).

Various techniques were described for the management of concealed penis including phallopexy or fixation of penile skin to the underlying Buck's fascia. This could be performed at one or two levels; the difference in the outcomes of the two types was not discussed widely in the literature. As a result, the goal of our research was to compare those two techniques.

Phallopexy (penile fixation) for the management of concealed penises was studied by many authors in the literature with good results, as described by Ci Zhang et al. in 2006. The overall success rate of phallopexy in our study was about 96.1% (12). Satisfactory results of this procedure with minimal post-operative complications were concluded by Aydin et al. (13) who recommended in their study phallopexy at 5 and 7 o'clock points in comparison to 3 and 9 o'clock points in our study, which are away from and protect the urethra.

An explanation of the importance of phallopexy was described by Alter et al. (14) who explained in their study that the actual cause of buried penis is the inadequate fixation of the penile dermis to Buck's fascia allowing the proximal telescoping of the corporeal bodies into the pubis and scrotum and consequently concluded that the golden step in the management of the such situation is by fixation of these two layers avoiding the urethra with the good post-operative outcome. Joseph (15) also has described good follow-up results of phallopexy in the management of buried penis after complete penile degloving and excision of the abnormally tethered dartos fascia.

Few studies comparing two levels vs. one level of phallopexy in the cases of the concealed penis in children were reported in the literature. Hence, we aimed to study this variable among a sample of children.

Our study observed a high success rate of phallopexy in the treatment of concealed penis (96.1%). However, it was observed that this was observed in two patients of those who had one level of phallopexy (2.2%) and in five patients of those who had two levels of phallopexy (5.6%) without an obvious explanation indicating that either technique could be used safely and successfully in such patients. Penile re-retraction was managed by educating the parents on how to pull the skin backward regularly with careful cleaning of any residue.

Penile skin edema was observed in 76 patients (42.2%); this may be attributed to penile degloving, which was performed in all patients before phallopexy, which could interrupt the lymphatic system, although this was regressed gradually during the early three post-operative months in all patients. Its presence was significantly lower in patients with lower body weight.

The procedure of penile fixation in the case of the congenitally buried penis is usually associated with minimal post-operative complications as observed in Valioulis et al. (16). The same was detected in our study, where only six patients (3.3%) developed post-operative skin congestion, which regressed gradually and spontaneously over the 1st post-operative month, and only one patient showed minimal skin infection; both of which resolved spontaneously.

## Conclusion

Phallopexy or fixation of the penile Buck's fascia to the subdermal level in the management of concealed penises has a high success rate in the avoidance of post-operative penile re-retraction with minimal post-operative complications.

Phallopexy could be performed at one level—at the penile base—or with the addition of another two stitches at the mid-penile level without making a significant difference with regard to post-operative penile re-retraction. So, we recommend the simpler one-level phallopexy with satisfactory results, as two-level phallopexy did not improve the results significantly, besides that this is demanding, time-consuming, and requires more resources.

### Limitations

One of the limitations of our study was the usage of PDS suturing material, so a longer follow-up period should be recommended in further studies comparing this absorbable material with a non-absorbable one. Another recommendation is a longer period of follow-up, more than one year, in a larger study to evaluate the delayed outcome of the procedure.

## Data Availability

The raw data supporting the conclusions of this article will be made available by the authors, without undue reservation.
